# Is There a Crucial Link Between Vitamin D Status and Inflammatory Response in Patients With COVID-19?

**DOI:** 10.3389/fimmu.2021.745713

**Published:** 2022-01-24

**Authors:** Federica Saponaro, Maria Franzini, Chukwuma Okoye, Rachele Antognoli, Beatrice Campi, Marco Scalese, Tommaso Neri, Laura Carrozzi, Fabio Monzani, Riccardo Zucchi, Alessandro Celi, Aldo Paolicchi, Alessandro Saba

**Affiliations:** ^1^ Department of Surgical, Medical, and Molecular Pathology and Critical Care Medicine, University of Pisa, Pisa, Italy; ^2^ Department of Translational Research and New Technologies in Medicine, University of Pisa, Pisa, Italy; ^3^ Department of Clinical and Experimental Medicine, University of Pisa, Pisa, Italy; ^4^ Institute of Clinical Physiology, National Council of Research, Pisa, Italy

**Keywords:** COVID-19, SARS-CoV-2, hypovitaminosis D, vitamin D, cytokine storm

## Abstract

**Background:**

Hypovitaminosis D has been suggested to play a possible role in coronavirus disease 2019 (COVID-19) infection.

**Methods:**

The aim of this study is to analyze the relationship between vitamin D status and a biochemical panel of inflammatory markers in a cohort of patients with COVID-19. A secondary endpoint was to evaluate the correlation between 25OHD levels and the severity of the disease. Ninety-three consecutive patients with COVID-19-related pneumonia were evaluated from March to May 2020 in two hospital units in Pisa, in whom biochemical inflammatory markers, 25OHD levels, P/F ratio at nadir during hospitalization, and complete clinical data were available.

**Results:**

Sixty-five percent of patients presented hypovitaminosis D (25OHD ≤ 20 ng/ml) and showed significantly higher IL-6 [20.8 (10.9–45.6) vs. 12.9 (8.7–21.1) pg/ml, *p* = 0.02], CRP [10.7 (4.2–19.2) vs. 5.9 (1.6–8.1) mg/dl, *p* = 0.003], TNF-α [8.9 (6.0–14.8) vs. 4.4 (1.5–10.6) pg/ml, *p* = 0.01], D-dimer [0.53 (0.25–0.72) vs. 0.22 (0.17–0.35) mg/l, *p* = 0.002], and IL-10 [3.7 (1.8–6.9) vs. 2.3 (0.5–5.8) pg/ml, *p* = 0.03]. A significant inverse correlation was found between 25OHD and all these markers, even adjusted for age and sex. Hypovitaminosis D was prevalent in patients with severe ARDS, compared with the other groups (75% vs. 68% vs. 55%, *p* < 0.001), and 25OHD levels were lower in non-survivor patients.

**Conclusions:**

The relationship between 25OHD levels and inflammatory markers suggests that vitamin D status needs to be taken into account in the management of these patients. If vitamin D is a marker of poor prognosis or a possible risk factor with beneficial effects from supplementation, this still needs to be elucidated.

## 1 Introduction

Since 2020, coronavirus disease 2019 (COVID-19), the infection due to the novel β coronavirus SARS-CoV-2, spread as a pandemic emergence showing a mutable phenotype ranging from asymptomatic to lethal severe acute respiratory syndrome.

Multiple biological and environmental factors have been investigated, to define the susceptibility to the diseases and precipitating events in morbidity and mortality. Among them, vitamin D status was proposed as a credible candidate, since hypovitaminosis D could be identified as a possible risk factor and a potential tool in COVID-19 prevention or ancillary treatment. This suggestion was initially based on indirect evidence, namely: i) the seasonal flare of COVID-19 which coincides with the nadir of vitamin D levels, ii) the association between hypovitaminosis D and pulmonary infections, and iii) the extraskeletal anti-inflammatory role of the active hormone vitamin D (1,25-dihydroxyvitamin D_3_ or calcitriol) which could be of benefit against the so-called “cytokine storm” (1).

From a clinical perspective, there is a strong epidemiological association between vitamin D deficiency and increased risk of infections, dysregulation of immune system, and autoimmune diseases (2). Vitamin D levels showed inverse correlation with the risk of multiple pulmonary injuries such as community acquired pneumonia, ARDS, sepsis, heart failure, and mortality secondary to pulmonary infections (3–7). Moreover, a systematic review and meta-analysis on more than 10,000 subjects demonstrated that vitamin D supplementation had a protective role in acute respiratory infections in adults (8).

In the last few months, many studies have been published on vitamin D and COVID-19, demonstrating the association between hypovitaminosis D and infection susceptibility and outcome. On the whole, a negative correlation was found between vitamin D levels and COVID-19 insurgence (9), and hypovitaminosis D showed a higher prevalence in patients with severe infection and it was predictive of hospitalization and mortality (10). Taken together, these data encouraged us to deepen our knowledge about the role of vitamin D in infection from SARS-CoV-2, and a lively debate is still open whether there is causality or no causality of this relationship.

A crucial point that still needs to be investigated is the involvement of poor vitamin D status in the inflammatory response by the host to SARS-CoV-2, which is directly correlated with morbidity and mortality. A recent review focused on the possible role of vitamin D as a modulator of the lung-centric inflammation burden of COVID-19, providing the rationale for further exploring the interplay between 25OHD levels, inflammatory marker/cytokine secretion, and outcome of the disease (11).

Therefore, the aim of this study is primarily to analyze the relationship between vitamin D status and a biochemical panel of inflammatory markers in a cohort of patients with COVID-19. As a secondary endpoint, we evaluated the correlation between 25OHD levels and the severity of the disease.

## 2 Materials and Methods

### 2.1 Study Design and Patients

This was a retrospective, observational study conducted on available serum samples from 93 consecutive patients with COVID-19-related pneumonia, admitted from March to May 2020 in two hospital units (*n* = 64 from a pulmonary unit and *n* = 29 from a geriatric unit) in Pisa. The two units were designated acute care units for patients with COVID-19-related pneumonia not requiring endotracheal intubation. COVID-19 infection was confirmed by a reverse transcription polymerase chain reaction test on a nasopharyngeal swab; the diagnosis of pneumonia was confirmed by the presence of consolidation(s) or ground-glass areas detected by chest computer tomography scans. Demographic data, clinical history, therapies, clinical data during hospitalization, routine blood data, blood coagulation parameters, arterial blood gas analysis, and imaging were available from the records of patients. In-hospital mortality and serious adverse events were also recorded.

The Institutional Review Board approved the study; all patients gave informed consent (protocol no. CEAVNO-2020-17241).

### 2.2 Biochemical Panel of Inflammatory Marker Measurement and Other Measurements

Samples from routine blood collection at hospital admission of all consecutive patients (*n* = 93) were stored at −20°C (protected from light to preserve vitamin D).

Analysis for cytokines (interleukins IL-1β, IL-6, and IL-10; tumor necrosis factor-α, TNF-α; monocyte chemotactic protein-1, MCPI-1/CCL2) was performed in the laboratory of the Clinical Pathology Unit of the University Hospital of Pisa by a fully automated ELISA processing system (DSX DINEX Technologies) using commercial ELISA assays according to the instructions of the manufacturer. The following kits were used: Human IL-1β Instant ELISA (eBioscience, Affymetrix), Human IL-6 Instant ELISA Kit (Invitrogen, Thermo Fisher Scientific), Human IL-10 Instant ELISA Kit (Invitrogen, Thermo Fisher Scientific), Human TNF-α Quantikine^®^ ELISA Kit (R&D Systems, Minneapolis, Canada), and Human CCL2/MCP-1 Quantikine^®^ ELISA Kit (R&D Systems, Bio-Techne). C-reactive protein (CRP) was measured by high-sensitive assay on BN II nephelometer (Siemens Healthineers). Quantification of D-dimer was obtained by the assay Vidas^®^ D-dimer exclusion (bioMérieux) performed in the Laboratory of Clinical Chemistry Unit (University Hospital, Pisa).

Hemogasanalysis was performed by GEM Premier 4000 Blood Gas Analyzer (Werfen, Spain), and gas exchange impairment was evaluated using arterial partial pressure of oxygen (PaO_2_) to fraction of inspired oxygen (FiO_2_) (P/F) (12). Patients were classified into three groups on the basis of the lowest value recorded during hospital stay (P/F nadir): patients with a P/F nadir ≥300 mmHg were categorized as “controls”, patients with a P/F nadir between 201 and 300 mmHg were categorized as mild acute respiratory distress syndrome (ARDS), and patients with a P/F nadir <200 mmHg were categorized as severe ARDS.

### 2.3 25OHD Measurement

25OHD levels were measured in the whole group (*n* = 93) of patients (blood samples were collected at baseline evaluation and the relative plasmas were stored at −20°C) with tandem mass spectrometry coupled to high-performance liquid chromatography (HPLC-MS-MS), using the MSMS VitD Kit from PerkinElmer (Waltham, MA, USA). It is well known that HPLC-MS-MS methods based on the isotope dilution technique usually suffer from very limited interfering effects and, consequently, offer a good quantification accuracy (13, 14).

#### 2.3.1 Instrumentation

The analytical device consisted of an Agilent 1290 Infinity UHPLC system (Santa Clara, CA, USA), including an autosampler, binary pump, and column oven, coupled to a Sciex QTRAP 6500+ that worked as a conventional triple quadrupole mass spectrometer (Concord, ON, Canada), and was equipped with an atmospheric pressure chemical ionization (APCI) source. The HPLC column was a PerkinElmer Brownlee Supra C18 3 µm, 50 × 2.1 mm, protected by a PerkinElmer Brownlee Supra C18 Guard Column.

#### 2.3.2 HPLC-MS-MS Conditions

The separation was carried out with gradient elution reversed-phase chromatography that made use of methanol + 0.1% formic acid as solvent A and water + 0.1% formic acid as solvent B. The MS method was based on positive ion multiple reaction monitoring (MRM) with the following quantifying transitions: 25OHD, *m*/*z* 401 > 159; 2H3-25OHD (IS), *m*/*z* 404 > 162; and 2H6-25OHD (used in calibrators and quality controls in place of 25OHD), *m*/*z* 407 > 159 (10).

#### 2.3.3 Sample Preparation

It consisted of conventional protein precipitation after the addition of a suitable amount of IS.

The intra-assay CV was specified by the producer and was <4.6%, while the inter-assay CV was <4.7%. Moreover, the HPLC-MS-MS method was validated by assaying 25OHD in a set of samples bought from DEQAS (the Vitamin D External Quality Assessment Scheme) and comparing the results with those from “true” DEQAS 25OHD values. Good performances were achieved since, as required, our laboratories had >75% of assessable results within ±25% of the true values from DEQAS.

### 2.4 Statistical Analysis

Continuous variables were expressed as mean values ± standard deviations or median values and interquartile range (IQR—25° and 75° quartiles) according to whether they were normally distributed or not. Categorical variables were expressed as the number of cases and percentages. Non-parametric or parametric tests were performed accordingly. Comparisons of qualitative data were performed using the chi-square test. For continuous variables, assessments of any possible differences between the different groups considered were performed using Mann–Whitney *U* tests. The association between PaO_2_/FiO_2_ subgroups and circulating levels of 25OHD levels was evaluated using the Jonckheere–Terpstra test for trend. Associations between serum 25OHD levels and inflammatory markers were analyzed using the Pearson correlation coefficient. *p*-values < 0.05 were considered as statistically significant. The statistical package SPSS (27.0) was used for analysis.

## 3 Results

### 3.1 Patients

A total of 93 consecutive patients were included in the study, and they were mainly males (*n* = 64, 68.9%) with a mean age of 68 ± 16 years (median 69, i.r. 57–80). According to the P/F ratio, 20 patients (21.5%) were classified as severe ARDS (P/F nadir < 200 mmHg) and 39 patients (41.9%) as mild ARDS (P/F nadir between 201 and 300 mmHg), while 34 patients (36.5%) presented a P/F ratio higher than 300 mmHg. Median follow-up time was 26 (13–29) days; during the study period, 15 patients (16.1%) died. Clinical and biochemical data of the whole group of patients are reported in **Table 1**.

**Table 1:** Clinical and biochemical characteristics of patients in the whole group of patients and according to 25OHD levels (cutoff 25OHD = 20 mg/ml).

	Whole group (*n* = 93)	25OHD ≤ 20 ng/ml (*n* = 61)	25OHD > 20 ng/ml (*n* = 32)	*p*
Age (years)^a^	68 ± 16	68 ± 16	66 ± 15	0.43
Gender, *n* (%)^b^				
– Males	64 (68.8)	44 (72.1)	20 (62.5)	0.98
– Females	29 (31.2)	17 (27.9)	12 (37.5)	
BMI (kg/m²)^a^	25.9 ± 4.5	25.8 ± 4.4	26.1 ± 4.9	0.95
Smoking habits, *n* (%)^b^				
– Current smoker	4 (4.3)	4 (6.6)	0 (0)	0.199
– Never smoker	66 (71.0)	38 (62.3)	28 (87.5)	
– Ex-smoker	23 (24.7)	19 (31.1)	4 (12.5)	
Major comorbidities, *n* (%)^b^				
– Diabetes mellitus	23 (24.7)	18 (29.5)	5 (15.6)	0.041
– Cardiovascular disease	27 (29.0)	19 (31.1)	8 (25.0)	0.382
– Cerebrovascular disease	9 (9.7)	5 (8.2)	4 (12.5)	0.595
– COPD	8 (8.6)	3 (4.9)	5 (15.6)	0.107
– Dementia	10 (10.7)	7 (11.5)	3 (9.4)	0.657
– Malignant disease	15 (16.1)	11 (18.0)	4 (12.5)	0.391
PaO_2_/FiO_2_ ratio at nadir (mmHg)^a^	176 ± 159	161.5 ± 146.7	237.5 ± 99	0.250
PaO_2_/FiO_2_: <200, *n* (%)^b^	20 (21.5)	15 (24.6)	5 (15.6)	
PaO_2_/FiO_2_: 200–300, *n* (%)^b^	39 (41.9)	27 (44.3)	12 (37.5)	0.296
PaO_2_/FiO_2_: >300, *n* (%)^b^	34 (36.5)	19 (31.1)	15 (46.9)	
25OHD levels (ng/ml)^c^	16.5 (7.9–23.3)	11.3 (6.4–16.4)	27.2 (23.2–33.1)	**0.001**
IL-6 (pg/ml)^c^	15.2 (9.8–32.8)	20.8 (10.9–45.6)	12.9 (8.7–21.1)	**0.028**
IL-1β (pg/ml)^c^	1.5 (1–2)	1.5 (0.9–2.2)	1.6 (1–1.9)	0.74
IL-10 (pg/ml)^c^	3.1 (1.2–6.3)	3.7 (1.8–6.9)	2.3 (0.5–5.8)	**0.03**
TNF-α (pg/ml)^c^	8.3 (3.7–13.8)	8.9 (6.0–14.8)	4.4 (1.5–10.6)	**0.01**
GM-CSF (pg/ml)^c^	2.2 (1.4–3.1)	2.2 (1.7–3.1)	1.9 (1.1–2.6)	0.61
MCP-1 (pg/ml)^c^	571 (407–833)	590 (448–975)	550 (407–736)	0.25
CRP (mg/dl)^c^	8 (2.7–14.9)	10.7 (4.20–19.16)	5.89 (1.63–8.15)	**0.003**
Ferritin (ng/ml)^c^	562 (300–1,113)	784.0 (321.0–1,376.0)	441.5 (296.0–899.5)	0.22
D-dimer (mg/l)^c^	0.37 (0.2–0.61)	0.53 (0.27–0.72)	0.22 (0.17–0.35)	**0.002**

a Biochemical data are expressed as mean ± SD.

b Data are expressed as n and % of the column (group).

c Biochemical data are expressed as median and interquartile range.

In bold: statistically significant differences.

### 3.2 Vitamin D Status and Association Between 25OHD Levels and Inflammatory Markers

Mean 25OHD was 17.3 ± 10.7 ng/ml, with a median of 16.5 ng/ml (i.r. 7.9–23.3). Eighty-nine percent of patients had 25OHD levels ≤30 ng/ml (*n* = 83), 65% (*n* = 61) had 25OHD levels ≤20 ng/ml, and 29% (*n* = 27) had 25OHD ≤10 ng/ml (severe vitamin D deficiency).

**Figure 1:**
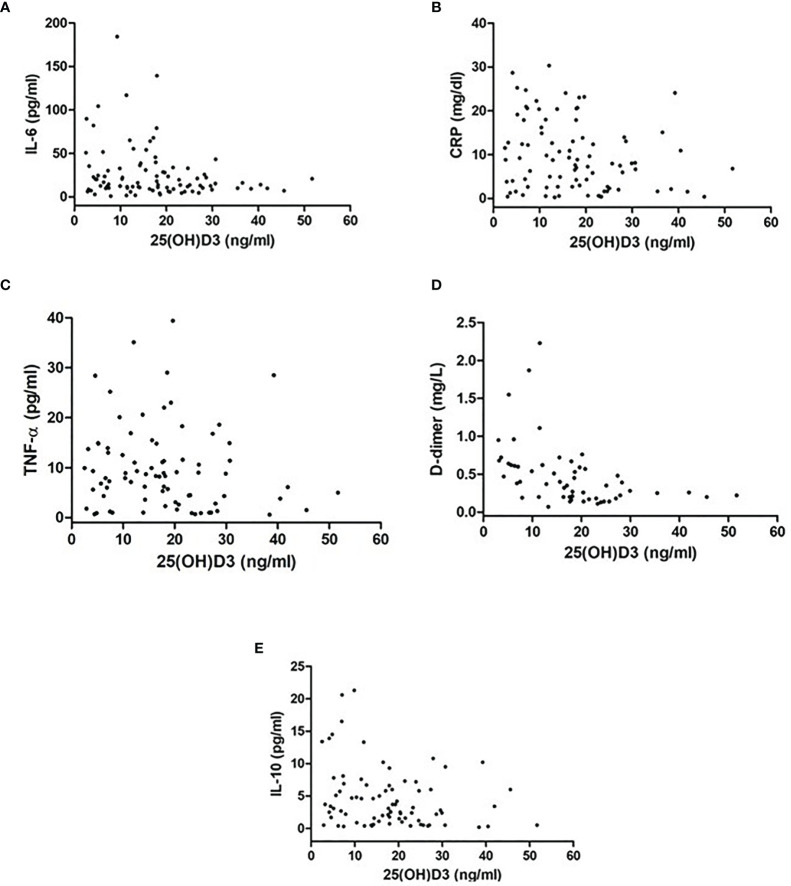
Difference in inflammatory markers [**(A)** IL-6, **(B)** CRP, **(C)** TNF-α, **(D)** D-dimer, **(E)** IL-10] between patients with 25OHD levels >20 ng/ml and those with 25OHD levels ≤20 ng/ml.

In the overall group, an inverse correlation was found between 25OHD and IL-6 (*r* = −0.22, *p* = 0.03), between 25OHD and CRP (*r* = −0.21, *p* = 0.04), between 25OHD and D-dimer (*r* = −0.43, *p* = 0.001), and between 25OHD and IL-10 (*r* = −0.25, *p* = 0.02), but not with TNF-α (*r* = −0.12, *p* = 0.1) (**Figures 1A–E**). These correlations remained statistically significant in a multiple linear regression analysis, adjusted for age and sex (*β* = −0.64, *p* = 0.04, IL-6; *β* = −0.17, *p* = 0.03, CRP; *β* = −0.017, *p* = 0.001, D-dimer; *β* = −0.11, *p* = 0.02, IL-10).

**Figure 2:**
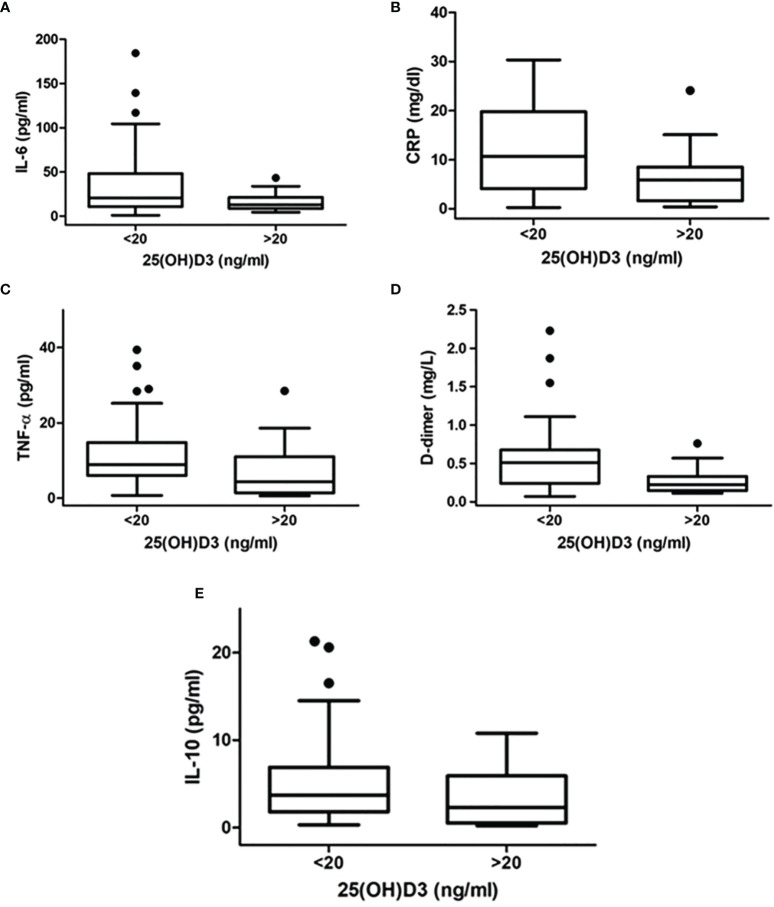
Correlation between inflammatory markers [**(A)** IL-6, **(B)** CRP, **(C)**TNF-alpha, **(D)** D-dimer, **(E)** IL-10] and 25OHD levels in patients with SARS-CoV-2.

Inflammatory markers were measured in all patients and compared between patients with 25OHD levels >20 ng/ml and those with 25OHD levels ≤20 ng/ml. The latter showed significantly higher IL-6 [20.8 (10.9–45.6) vs. 12.9 (8.7–21.1) pg/ml, *p* = 0.02], CRP [10.7 (4.2–19.2) vs. 5.9 (1.6–8.1) mg/dl, *p* = 0.003], TNF-α [8.9 (6.0–14.8) vs. 4.4 (1.5–10.6) pg/ml, *p* = 0.01], D-dimer [0.53 (0.25–0.72) vs. 0.22 (0.17–0.35) mg/l, *p* = 0.002], and IL-10 [3.7 (1.8–6.9) vs. 2.3 (0.5–5.8) pg/ml, *p* = 0.03] (**Figures 2A–E**).

### 3.3 Vitamin D Status and Severity of the Disease

**Figure 3:**
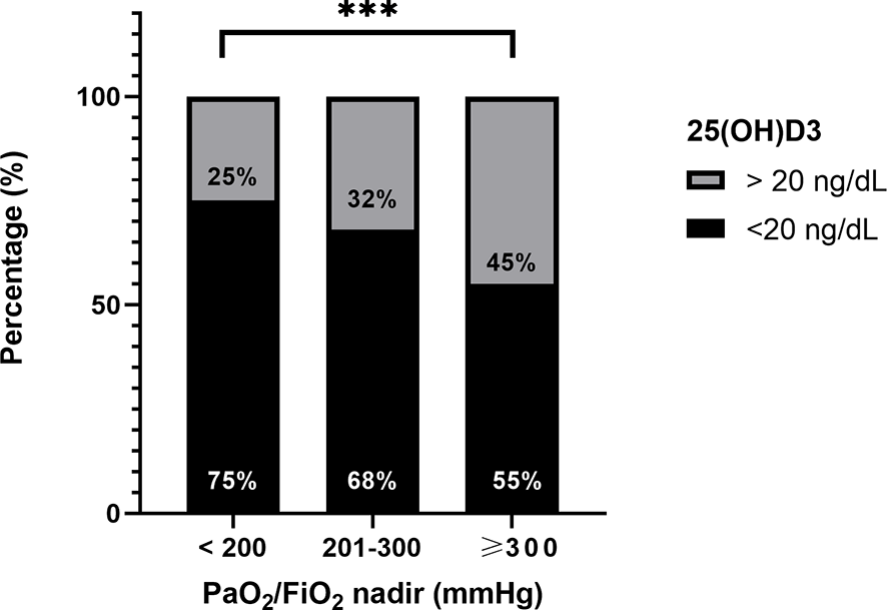
Difference in proportion of patients with vitamin D insufficiency (25OHD levels ≤ 20 ng/ml) among patients with PaO_2_/FiO_2_ <200, 201–300, and ≥300 mmHg. ***p < 0.0001.

The proportion of patients with vitamin D insufficiency (25OHD levels ≤ 20 ng/ml) was significantly higher in patients with PaO_2_/FiO_2_ <200 mmHg, compared with those patients with PaO_2_/FiO_2_ 201–300 mmHg and PaO_2_/FiO_2_ ≥300 mmHg (75% vs. 68% vs. 55%, *p* < 0.001) (**Figure 3**).

**Figure 4:**
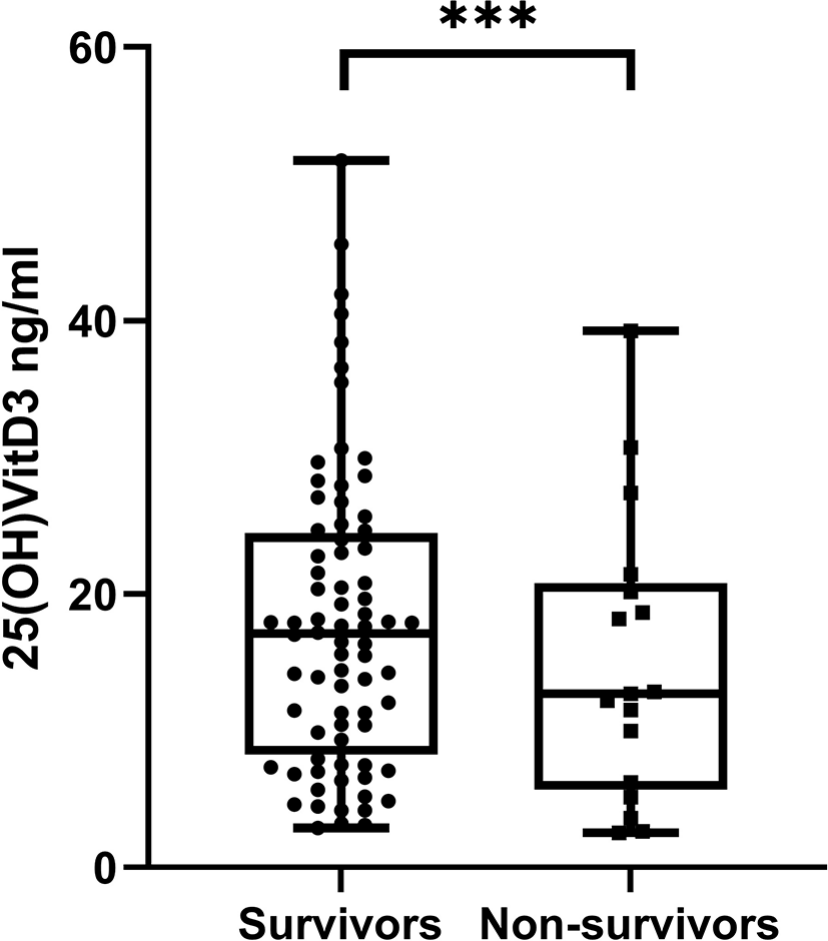
Difference in 25OHD levels between survivors and non-survivors patients. ***p < 0.0001.

Finally, we evaluated the difference between 25OHD levels in survivor patients and non-survivors, and we found that 25OHD was significantly lower in the latter compared with the former [median 17.0 (8.6–24.3) vs. 12.7 (5.4–21.1), *p* < 0.000] (**Figure 4**).

## 4 Discussion

In the last one year and half, the world has been upset by the COVID-19 emergency and scientific research has been pushed to shed light on risk factors for the infection and progression to fatal outcome. Vitamin D deficiency and COVID-19 binomial is still hotly debated in the literature, but the exact role of this hormone in the complex scenario of the pandemic infection needs to be elucidated. Solid evidence sustains that hypovitaminosis D is a common finding in COVID-19 patients, and this has been interpreted alternatively as a marker of poor health and a susceptibility factor or as a possible modifiable risk factor with physiopathological implications and benefits from supplementation. Further multicentric international randomized clinical trials, which are still ongoing, would probably resolve the question. As a matter of fact, a bidirectional correlation has been suggested between hypovitaminosis D and a major feature of COVID-19, namely, the potent inflammatory response of the host.

This study aims to contribute to the field focusing on the relationship between vitamin D and immune system activation during COVID-19. To study that, we performed a systematic evaluation of multiple inflammatory markers all measured at the same moment at the beginning of hospitalization, we accurately measured 25OHD levels by the gold standard methodology LC-MS-MS, and we used P/F at nadir as the standardized index of the severity of the disease.

Among several measured inflammatory markers, which best depict the inflammatory status of patients with COVID-19 infection, we found high levels of IL-6, CRP, TNF-α, IL-10, and D-dimer in patients with hypovitaminosis D. Further data revealed that there was an inverse correlation between these markers and 25OHD levels, even when adjusted for age and sex. These findings allow a general overview on the cytokine response during viral infection from SARS-CoV-2 and suggest an interplay between inflammatory response and vitamin D metabolism.

Other studies showed an association between inflammation and vitamin D status, even if they focused only on one or two markers individually. In agreement with our results, Jain et al. described a cohort of 154 patients divided into two groups: asymptomatic COVID-19 patients and patients with severe disease and ICU requirement. In both groups, patients with 25OHD <20 ng/ml had higher levels of IL-6 and TNF-α, even if the latter did not reach the threshold of statistical significance (15). Our data regarding IL-6 are also in agreement with those of Radujkovic et al., who evaluated 185 patients and found that median IL-6 measured at the moment of hospitalization was significantly higher in patients with severe vitamin D deficiency (i.e., 25OHD < 12 ng/ml).

In our study, CRP was significantly higher in patients with hypovitaminosis D, and an inverse correlation between 25OHD levels and CRP was observed. This is consistent with the role of CRP in COVID-19 evolution: recent investigations demonstrated that CRP is correlated with lung lesion appearance and with the severity of the disease (16). Moreover, CRP has been shown to increase even before CT findings and could predict the poor outcome of COVID-19 (17). Consistent with our data, in a cohort of 235 COVID-19 patients, Maghboli et al. showed that CRP levels were lower in patients with 25OHD >30 ng/ml, and in patients with hypovitaminosis D, the relative risks to show high CRP (>40 mg/l) and to have a severe disease were 1.7 and 1.59, respectively. In another study by Daneshkhah et al., high levels of CRP were associated with hypovitaminosis D and severe outcome of infection by SARS-CoV-2 (18). A few studies also evaluated the relationship between D-dimer levels and 25OHD. In agreement with our data, the retrospective study by Demir et al. on 227 COVID-19 patients showed that D-dimer was higher in the presence of low 25OHD levels (19). Similar results were reported by Giannini et al. in a cohort of 91 patients (20).

The focus on the inflammatory status of patients with SARS-CoV-2 infection is built upon the knowledge that the inflammatory response of the host itself has a pivotal role in COVID-19 severity and mortality (21).

SARS-CoV-2 is able to infect nasal, bronchial, and alveolar epithelial cells, with major burden in the lungs, as established by studies on autopsies, where alveolar destruction, presence of thrombi, and severe local inflammation have been found. However, the morbidity and mortality of COVID-19 disease is related to the dysregulation of the immune system of the host, with a decreased capacity to deal with the virus and an excessive release of cytokines and other inflammation mediators (11, 22).

COVID-19 is indeed a systemic disease, with multiorgan involvement, including also extensive microvascular damage, not limited to the lungs, general cardiovascular injury, and an aggressive inflammatory response which triggers a negative prognosis (23–25). This condition is due to a dysregulation of immune response after rapid viral replication, with a massive and turbulent recruitment of inflammatory cells and cytokine release, alteration of vascular and alveolar barrier, and finally, development of fatal ARDS (26, 27). In patients with severe COVID-19, high levels of circulating IL-6, IL-1β, INFγ, and TNF-α have been reported, together with an unbalanced white cell formula, consisting of high neutrophils and decreased CD4^+^ and CD8^+^ T cells (27–30).

The contemporary evaluation of multiple inflammatory markers in our study, all converging to the same direction, suggests that the poorest is the vitamin D condition and the highest is the inflammation response. The moderate blood levels of cytokines in our population of patients with COVID-19 not requiring endotracheal intubation are probably due to the moderate severity of the disease.

Unfortunately, the retrospective nature of this study does not allow us to infer if hypovitaminosis D has a causal effect on the immune system or a reverse causality is still possible. Indeed, some studies have shown that vitamin D acts as an “acute phase reactant” being reduced during acute and chronic diseases for several reasons (31). Among them, the acute inflammation status itself, a reduction of vitamin D binding proteins, and hemodilution could be responsible for 25OHD decrease (32). If this is the case, vitamin D status could be at least a marker of poor prognosis and severity for COVID-19.

On the other hand, it is noteworthy that other studies have confirmed the relationship between 25OHD levels measured before the occurrence of SARS-CoV-2 infection and severity of the disease (33–35), as claimed also by another very recent Italian study by Campi et al., whose results are similar to ours (36). In this case, a reverse causality is unlikely. Moreover, some considerations regarding the physiological role of active metabolites of vitamin D on the immune system need to be taken into account.

A pathophysiological role for vitamin D deficiency in COVID-19 is sustained by the established involvement of the vitamin D active metabolite calcitriol 1,25(OH)_2_D in immune system regulation and modulation (37, 38).

Different from the classical endocrine role of 1,25(OH)_2_D in calcium homeostasis, the effect of active vitamin D either on innate and adaptive immune response seems to be mediated by a paracrine and intracrine mechanism, related to the local presence of the enzyme hydroxylase CYP27B1 (39, 40). With regard to the innate immune response, 1,25(OH)_2_D can enhance antiviral defense by several mechanisms (38, 41, 42), and 1,25(OH)_2_D is also able to modulate acquired immune responses, decreasing the expression of MHC class II and co-signaling molecules on antigen-presenting cells, reducing TH1 and TH17 cell activity, and upregulating regulatory T cells (43).

Finally, 1,25(OH)_2_D has a role in reducing tissue factor activity, as well as prothrombotic factors and proinflammatory signals in blood vessel, which contribute to microvascular disease involvement of COVID-19 (44). Consistently, hypovitaminosis D has been also correlated with cardiovascular impairment and prothrombotic and cerebrovascular events, in the context of well-known cardiovascular extraskeletal effects (45–51).

The secondary endpoint of this study was the evaluation of the relationship between vitamin D levels and COVID-19 severity and outcome, and we showed that vitamin D levels were lower in the group with more severe disease and in non-survivor patients. The raising of the inflammatory markers themselves is correlated with a worse prognosis in patients with COVID-19, as already known in the literature and also confirmed in this dataset (data not shown), confirming that cytokine production is predictive of poor fate. However, we focused our analysis on vitamin D and the outcome of the patients, to evaluate the possible “protective role” of this hormone and not only the predictive value of hypovitaminosis D.

A strength of the present study is the use of the arterial partial pressure of oxygen (PaO_2_) to fraction of inspired oxygen (FiO_2_) (P/F) measured as the lowest value recorded during hospital stay to stratify the severity of disease of the patients. A very recent systematic review of the literature included nine studies with the specific aim of finding a relationship between COVID-19 infection and/or prognosis and vitamin D levels: seven studies confirmed this link and four studies in particular showed an association between hypovitaminosis D and COVID-19 poor outcome and mortality (52). It is noteworthy that in the two studies that could not find an association, 25OHD levels had been measured many months before hospitalization and probably did not reflect the vitamin D status at the moment of infection (33, 34). Notably, in the investigations by Mendy et al. (on 689 COVID-19-positive patients), vitamin D deficiency was shown to be associated with COVID-19 severity and admission to ICU with an OR of 1.95 (95% CI 1.07–3.56) and 2.55 (95% CI 1.28–5.08), respectively. Hospitalization length was also related to vitamin D deficiency (53). In a retrospective Italian study by Carpagnano et al., 42 COVID-19 patients were evaluated: in agreement with our data, the majority of patients presented 25OHD levels <30 ng/ml (81% vs. 89% in the present study). Severe vitamin D deficiency or 25OHD levels <10 ng/ml were associated with 50% of mortality after 10 days of hospitalization, while 25OHD >10 ng/ml was associated with only 5% of mortality probability (*p* = 0.019) (54). In another recent meta-analysis including six studies and 376 COVID-19-positive patients, poor prognosis defined as severe symptoms, ICU admission, or death was present in 150 patients who also showed significantly lower levels of 25OHD, compared with patients with good prognosis (55). Finally, Campi et al. found that a 1-ng/ml increase in 25OHD levels was associated with a decrease of risk of death of 4% (36). On the other hand, a different approach using a large Mendelian study did not find a genetic association between vitamin D levels and susceptibility or mortality for COVID-19 (56).

Only further data from ongoing RCTs (57–59) are expected to assess the crucial question whether vitamin D supplementation can modify outcome and mortality in COVID-19 patients. Right now, only few encouraging data are present: in the retrospective Giannini study, the administration of a bolus of 400,000 UI oral cholecalciferol was associated with a reduced risk of incidence of ICU/death considering also the comorbidity burden of the patients (20). Moreover, in a small prospective Spanish study of 76 patients with SARS-CoV2, the administration of calcifediol significantly reduced ICU necessity compared with untreated patients (OR 0.03, 95% CI 0.003–0.25). In Italy, a recent expert consensus from the Glucocorticoid-Induced Osteoporosis and Skeletal Endocrinology Group (G.I.O.S.E.G.) recommended at least the measurement of 25OHD levels in those patients with comorbidities or risk factors for either hypovitaminosis D or COVID-19 (diabetes, obesity, pulmonary diseases) and also recommended supplementation for all patients with hypovitaminosis D (60).

In summary, our study shows the following strengths: i) simultaneous evaluation of several proinflammatory markers in patients with COVID-19, ii) direct correlation between these markers and 25OHD levels, iii) 25OHD levels measured by LC-MS-MS as the gold standard, and iv) evaluation of the severity of the disease by an objective outcome marker.

Our study has also some limitations. Due to the possibility to collect data from patients hospitalized for COVID-19, this is a retrospective study, and we have no data regarding 25OHD levels before hospitalization. Therefore, we cannot answer the question whether there is “causality or reverse causality”. Patients were enrolled during the first wave of COVID-19 and probably there were some differences with the clinical behavior in the subsequent waves. TNF-α when considered as a continuous variable had no statistically significant correlation with 25OHD levels, even if it was significantly different between patients with and without hypovitaminosis. We do not have a clear explanation to these data, probably due to a non-linear correlation between TNF-α and vitamin D.

## 5 Conclusions

In conclusion, the present study focuses on the link between vitamin D and inflammatory response, which is in turn correlated with the severity of COVID-19. The observed relationship suggests that vitamin D status needs to be taken into account in the clinical management of patients with SARS-CoV2 infection as a marker of poor prognosis and complications or as a possible modifiable risk factor for susceptibility. Further studies are needed, but the whole body of evidence in the literature urgently claims to continue to challenge the research in understanding the binomial vitamin D and COVID-19.

## Data Availability Statement

The datasets presented in this article are not readily available because they are the property of PISA COVID GROUP. Requests to access the datasets should be directed to PROF. M.FALCONE, marco.falcone@unipi.it.

## Ethics Statement

The studies involving human participants were reviewed and approved by the CEAVNO Institutional Review Board (protocol no. CEAVNO-2020-17241). The patients/participants provided their written informed consent to participate in this study.

## Author Contributions

FS with the help of MF and CO was responsible for the data collection and analysis and the writing of the manuscript draft. AS and AP were responsible for the original idea and supervision. AC helped in interpreting the clinical and functional data. FM was responsible for the clinical database of the geriatric section. LC and RZ were responsible for the supervision and writing of the manuscript draft. RA and TN helped in collecting the data of the patients. BC helped in performing the sample analysis. MS was responsible for the statistical analysis. All authors contributed to the article and approved the submitted version.

## Conflict of Interest

The authors declare that the research was conducted in the absence of any commercial or financial relationships that could be construed as a potential conflict of interest.

## Publisher’s Note

All claims expressed in this article are solely those of the authors and do not necessarily represent those of their affiliated organizations, or those of the publisher, the editors and the reviewers. Any product that may be evaluated in this article, or claim that may be made by its manufacturer, is not guaranteed or endorsed by the publisher.
